# Intrinsic molecular signature of breast cancer in a population-based cohort of 412 patients

**DOI:** 10.1186/bcr1517

**Published:** 2006-07-17

**Authors:** Stefano Calza, Per Hall, Gert Auer, Judith Bjöhle, Sigrid Klaar, Ulrike Kronenwett, Edison T Liu, Lance Miller, Alexander Ploner, Johanna Smeds, Jonas Bergh, Yudi Pawitan

**Affiliations:** 1 Department of Medical Epidemiology and Biostatistics, Karolinska Institutet, Nobel väg 12A, SE-171 77 Stockholm, Sweden; 2 Department of Oncology and Pathology, Cancer Center Karolinska, Radiumhemmet, Karolinska Institutet and University Hospital, Solna SE-171 76 Stockholm, Sweden; 3 Genome Institute of Singapore, 60 Biopolis Street, #02-01, Genome, 138672 Singapore; 4 Section of Medical Statistics and Biometry, Department of Biotechnologies and Biomedical Sciences, Viale Europa 11, 25123 Brescia, University of Brescia, Italy

## Abstract

**Background:**

Molecular markers and the rich biological information they contain have great potential for cancer diagnosis, prognostication and therapy prediction. So far, however, they have not superseded routine histopathology and staging criteria, partly because the few studies performed on molecular subtyping have had little validation and limited clinical characterization.

**Methods:**

We obtained gene expression and clinical data for 412 breast cancers obtained from population-based cohorts of patients from Stockholm and Uppsala, Sweden. Using the intrinsic set of approximately 500 genes derived in the Norway/Stanford breast cancer data, we validated the existence of five molecular subtypes – basal-like, ERBB2, luminal A/B and normal-like – and characterized these subtypes extensively with the use of conventional clinical variables.

**Results:**

We found an overall 77.5% concordance between the centroid prediction of the Swedish cohort by using the Norway/Stanford signature and the *k*-means clustering performed internally within the Swedish cohort. The highest rate of discordant assignments occurred between the luminal A and luminal B subtypes and between the luminal B and ERBB2 subtypes. The subtypes varied significantly in terms of grade (*p *< 0.001), p53 mutation (*p *< 0.001) and genomic instability (*p *= 0.01), but surprisingly there was little difference in lymph-node metastasis (*p *= 0.31). Furthermore, current users of hormone-replacement therapy were strikingly over-represented in the normal-like subgroup (*p *< 0.001). Separate analyses of the patients who received endocrine therapy and those who did not receive any adjuvant therapy supported the previous hypothesis that the basal-like subtype responded to adjuvant treatment, whereas the ERBB2 and luminal B subtypes were poor responders.

**Conclusion:**

We found that the intrinsic molecular subtypes of breast cancer are broadly present in a diverse collection of patients from a population-based cohort in Sweden. The intrinsic gene set, originally selected to reveal stable tumor characteristics, was shown to have a strong correlation with progression-related properties such as grade, p53 mutation and genomic instability.

## Introduction

A significant reduction of disease burden from breast cancer can be achieved by a better characterization of its potentially fatal forms. Spread of the disease, cell structure, differentiation and growth patterns have been used to classify tumors and to make a clinically relevant assessment of their metastatic potential. With an increasing use of screening programs, more early-stage tumors are diagnosed, creating a need for new tools for prognostication and therapy prediction. Standard clinical factors often lead to undertreatment and overtreatment of large patient cohorts. The classical factors also offer little or no insight into the pathogenesis and biological separation of distinct subtypes of human breast cancers.

The emergence of powerful molecular techniques allowing genome-wide analysis has the potential to improve tumor classification markedly. Using microarray-based gene expression analyses, Sorlie and colleagues [1] described five subtypes of breast cancer, roughly dividing estrogen receptor (ER)-negative and ER-positive tumors into two and three subclasses, respectively. Molecular techniques are growing more and more important as diagnostic and prognostic tools, but they have not yet come into routine clinical practice. One reason could be there had been limited validation [2] as well as limited clinical description [1] of the molecular subtypes. Validation is often difficult in this context, because results are influenced not only by patient selection but also by the choice of methods used to analyze gene expression data.

Consequently, our aims for this study were, first, to validate the previously derived molecular subtypes on a large population-based cohort of 412 patients in Sweden, and second, to characterize the genetic subtypes with the use of routine clinical variables, and to achieve these goals by using transparent and widely accepted analysis methods.

## Materials and methods

The total study population consisted of 412 patients for whom we had quality-controlled RNA expression microarray data, including 159 patients from Stockholm and 253 from Uppsala. The Stockholm subcohort includes all breast cancer patients that were operated at the Karolinska Hospital from 1 January 1994 to 31 December 1996 identified from the population-based Stockholm–Gotland breast cancer registry established in 1976. The ethical committee at the Karolinska University Hospital approved this microarray expression project. This cohort has previously been described in detail [3,4].

### Study population

The Stockholm–Gotland Breast Cancer Registry, supplemented with patient records, were examined for information on tumor size, number of retrieved and metastatic axillary lymph nodes, hormonal receptor status, distant metastases, site and date of relapse, initial therapy, therapy for possible recurrences, and date and cause of death. Tamoxifen and/or goserelin were normally used for hormonal treatment, whereas mostly six courses of intravenous cyclophosphamide–methotrexate–5-fluorouracil (CMF) on days 1 and 8 were used as adjuvant chemotherapy, except for high-risk patients. After primary therapy, patients were recommended to have regular clinical examinations and yearly mammograms, in addition to laboratory and X-ray tests guided by clinical signs and symptoms. Patients followed-up outside the Karolinska Hospital were tracked by using their unique Swedish personal identification number. There was no loss to follow-up.

Relapse site, date of relapse, relapse therapy, and date of death were ascertained until May 2002. The average follow-up was 6.1 years. Cause of death was coded as death due to breast cancer (including those with distant metastases, but dying from other causes), death due to other malignancies, and death due to non-malignant disorders. Subsequent primary malignancies were identified through the population-based Swedish Cancer Registry.

A second data source (referred to as Uppsala subcohort) consists of a population-based cohort of primary breast cancer patients receiving primary therapy from 1987 to 1989 in the county of Uppsala, Sweden. From the initial set of 315 patients, representing 65% of all breast cancer patients in the Uppsala county during these years, we were able to obtain quality-controlled RNA expression profiles from 253 frozen tumors. The patients were followed until December 1999. Overall, 76 lymph-node-positive and 26 lymph-node-negative patients received adjuvant therapy, mostly intravenous CMF-based therapy or adjuvant tamoxifen, whereas 130 patients did not receive adjuvant therapy. The ethical committee at the Karolinska Institutet approved this RNA expression study, and consent was obtained from each patient for the use of the biological tissues.

### RNA preparation

RNA was prepared with an RNeasy spin column kit (Qiagen, Valencia, CA, USA). Frozen tumors were cut into minute pieces and homogenized for 40 seconds in RNeasy lysis buffer. Proteinase K was added [5] and the samples were incubated for 10 minutes at 55°C, followed by centrifugation and the addition of ethanol. After the transfer into RNeasy columns, DNase was added to increase RNA quality. RNA quality was assessed with an Agilent 2100 bioanalyzer (Agilent Technologies, Rockville, MD, USA). The material was stored at -70°C. The amount of RNA for each probe preparation varied between 2 and 5 μg. First-strand cDNA synthesis was generated by using a T7-linked oligo(dT) primer, followed by second-strand synthesis. The *in vitro *transcription reactions were performed in batches to generate biotinylated cRNA targets, which were subsequently chemically fragmented at 95°C for 35 minutes.

Fragmented, biotinylated cRNA (10 μg) was hybridized at 45°C for 16 hours to Affymetrix high-density oligonucleotide HG-U133AB gene-chip arrays. The arrays were then washed and stained with streptavidin–phycoerythrin (10 μg/ml). Signal amplification was achieved with a biotinylated anti-streptavidin antibody. The scanned images were inspected for the presence of artifacts. In case of defects, the hybridization procedure was repeated. Expression values and detection calls were computed from raw data by following the procedures outlined for the Affymetrix MAS 5.0 analysis software [6]. Global mean normalization of MAS 5.0 expression was performed on a logarithmic scale to reduce differences in chip intensity [7].

A sample was either relabeled and the hybridization repeated, or excluded from further analysis if a scaling factor greater than four was necessary, if less than 30% present calls were found, or if the squared multiple correlation coefficient of the expression data on the array to all other arrays was below 0.6.

### Microarray data analysis and clustering methods

A publicly available set of 122 tumor samples [2] was downloaded from the Stanford Microarray Database [8]. Mean-normalized log_2 _ratios of expression values were used in the analysis. The so-called 'intrinsic' gene list as described by Sorlie and colleagues [2] was used to select genes of interest. CloneID and Unigene ID were matched by using the Stanford SOURCE Search website [9]. A total of 516 genes out of the original 552 had valid Unigene IDs. We excluded 32 genes assigned to multiple Unigene clusters and based our analyses on the resulting 484 genes with unique Unigene IDs.

Gene expression data from HG-U133AB Affymetrix chips were available for the Swedish samples. Matching between the Norway/Stanford data and the two Swedish data sets was achieved by using Unigene IDs build 181. In all, there were 465 genes with unique Unigene IDs that matched the Stanford intrinsic gene set.

Genes were median-centered and subtypes were defined by a hierarchical clustering of the Norway/Stanford data, using uncentered correlation as a distance metric and an average-linkage clustering algorithm. This step attempts only to reproduce the results of Sorlie and colleagues [2], so we expect to find their five subtypes (basal-like, ERBB2, luminal A, luminal B and normal-like). The profile of a subtype – the so-called 'centroid' – was computed by averaging expression values across each gene within each of the subtypes. Thus, the centroid simply represents the average gene expression over the tumors in a subtype. To obtain better defined and more homogeneous genetic profiles, we used only representative tumor samples in defining the centroids; representative was defined as having Pearson correlation of at least 0.2 with all other samples in the same subtype class.

### Consistency of clustering results

We then assigned the Swedish tumor samples to the five subtypes discovered in the Norway/Stanford data. This was achieved by calculating the Pearson correlation between each sample and each of the five centroids. Samples were then assigned to the subtype of the centroid with the largest correlation coefficient; we will refer to this procedure as 'centroid prediction'. If the correlations with all five centroids were below 0.1, a sample was labeled as 'unclassified'.

The centroid prediction of tumor samples to specific subtypes as described above represents a classification procedure, using Norway/Stanford data as the training set; that is, we forced the samples to fit into one of five prespecified categories. We evaluated whether this class allocation was consistent with the *k*-means clustering of the samples themselves. If there had been little consistency between the centroid prediction and the *k*-means clustering, then the class assignment would have been spurious. In this analysis we excluded the unclassified samples.

In brief, samples were scaled to have a mean of 0 and a standard deviation of 1, and clustered with a *k*-means algorithm with the number of cluster centers set to *k *= 5. The resulting five clusters were matched to the centroid-predicted labeling in such a manner as to maximize their agreement. The scaling step was included to make the Euclidean distances used in the *k*-means algorithm more similar to the correlation distances used in the centroid prediction. To allow for potential systematic differences in sample handling, the Stockholm and Uppsala cohorts were clustered separately. Concordance is expressed as the overall percentage of samples classified in the same group by the centroid prediction and *k*-means clustering methods. Intuitively, if this procedure is applied to randomly generated data, there will be little concordance between the two methods. Any discordant assignment indicates some similarity or overlapping characteristics between two subtypes.

### Clinical and survival data analysis

The genetically derived subtypes were characterized on a number of clinical characteristics, including age at diagnosis, tumor size, lymph-node status, grade and receptor status, and use of hormone replacement therapy (HRT). Mutation of p53 was ascertained by complete sequencing of the *p53 *gene [10]. Genomic instability was assessed on the basis of image cytometric data of DNA content; tumors were classified into diploid, aneuploid or tetraploid groups, and also whether they were genomically stable or unstable according the stemline-scatter index (SSI) developed by Kronenwett and colleagues [11]. SSI is the sum of the percentages of cells with DNA content values in the S-phase region (S phase), plus the percentage of cells with DNA content values exceeding twice the modal value plus 1*c *(G2 exceeding rate, or G2 Exc), plus the coefficient of variation of the tumor stemline. A genomically unstable tumor is defined to have an SSI of more than 8.8, indicating a significant cell-to-cell variation in DNA content.

Finally we performed a survival analysis, considering as outcome both the relapse-free interval and the occurrence of death due to either breast cancer or distant metastases. Given their uncertain biological interpretation, 43 unclassified samples (*n *= 20 in Stockholm and *n *= 23 in Uppsala) were excluded from the analysis. To obtain homogeneously defined treatment groups, we also excluded from the Stockholm cohort any patients with no adjuvant therapy and those treated with chemotherapy. In Stockholm, post-operative adjuvant therapy was a standard procedure; patients who did not receive the therapy either denied it or were considered too unfit for treatment. In contrast, the adjuvant-untreated patients in Uppsala were part of a clinical trial. We analyzed separately the endocrine-treated patients (*n *= 171) and those who did not receive any adjuvant therapy (*n *= 130). Among the endocrine-treated patients there were 16 patients who also received chemotherapy. To maintain power, these were kept in the analysis. Univariate Cox proportional models considered as a unique predictor variable the tumor subtype. All of the statistical analysis was performed with R [12] and Bioconductor [13], whereas clusters were displayed with treeview [14].

## Results

### Norway/Stanford data

**Figure 1:**
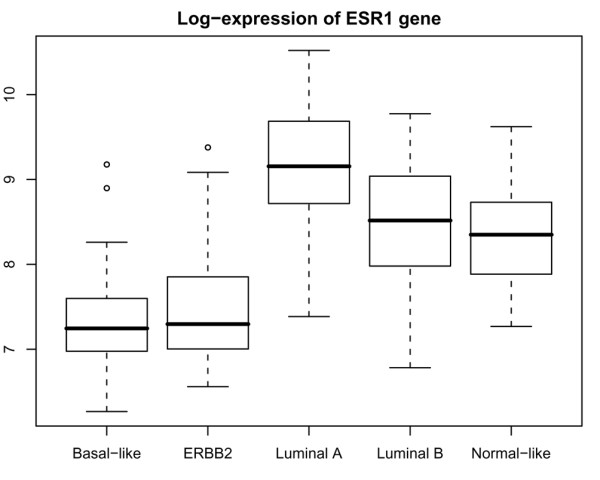
Logarithmic expression of the *ESR1 *gene. Boxplots are shown of *ESR1 *gene expression values (log_2 _transformed) against subtype in both Swedish cohorts.

**Figure 2:**
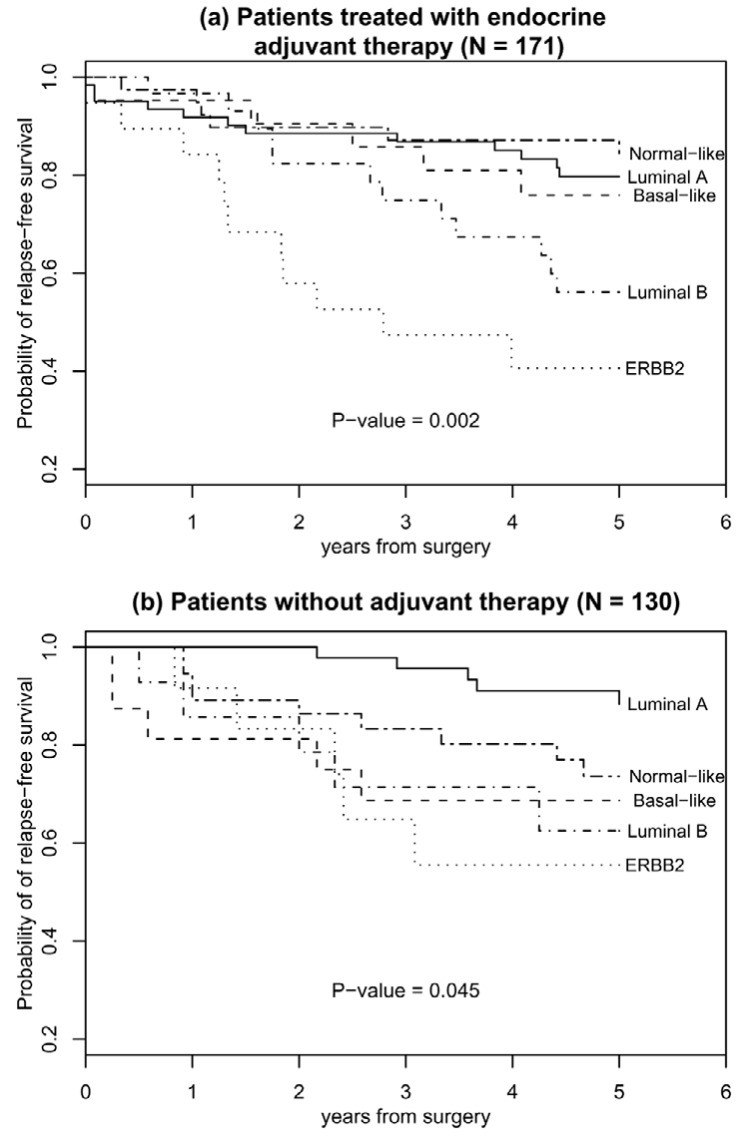
Kaplan-Meier relapse-free survival curves. Survival curves are shown for the tumor subtypes in **(a) **patients with endocrine therapy and **(b) **patients without any adjuvant therapy. The *p *value, computed with Cox regression, is for simultaneous comparison of all the curves.

We first analyzed the Norway/Stanford data to establish the previous molecular subtypes: basal-like, ERBB2, luminal A, luminal B and normal-like. This was achieved by hierarchical clustering of the 122 samples from Norway/Stanford based on 516 genes (Figure 1S in Additional file 1). The five subgroups reported by Sorlie and colleagues [2] were identifiable, and 93% of the tumors clustered in the same way. Moreover, the clusters were characterized by the same main genes as previously described, including *ERBB2*, *GRB7 *and *PPARBP *for the ERBB2 cluster, *CXCL1*, *KRT5*, *KRT17 *and *TRIM29 *for the basal-like cluster, *ESR1*, *GATA3 *and *SCUBE2 XBP1 *for luminal A, *SQLE*, *GGH *and *LAPTM48B *for luminal B, and *PIK3R1 *and *AKR1C1 *for normal-like samples (Figure 2S in Additional file 1).

Subtype centroids were then defined by including tumors with a pairwise correlation higher than 0.2, as shown in Table 1. All the samples considered in the centroids were also part of the centroids defined by Sorlie and colleagues [2], except for the ERBB2 centroid, where three samples out of seven were unclassified by Sorlie and colleagues [2]. The centroids are marked in a dendrogram in Figure 1S in Additional file 1.

### Swedish data

Using centroid prediction towards the five subtypes in the Norway/Stanford data, 59 samples were classified as basal-like, 43 as ERBB2, 122 as luminal A, 54 as luminal B and 91 as normal-like tumors (Table 1). A total of 43 samples showed a correlation of less than 0.1 with any centroid and were consequently put into the unclassified group.

The data were then clustered by using the same hierarchical clustering procedure as for the Norway/Stanford data; the dendrograms are displayed in Figures 3S and 4S in Additional file 1. As reported previously [15,16], the main separation was between tumors overexpressing estrogen-related genes, previously labeled as normal-like and luminal A/B, versus tumors negative for these genes, including the basal-like and ERBB2 subtypes. However, in contrast with the Norway/Stanford and Stockholm data, almost all Uppsala samples classified as luminal B (28 of 31) clustered in the same main branch as basal-like and ERBB2 clusters, whereas normal-like tumors were located close to the luminal A group.

Most samples associated with the basal-like subtype clustered together, showing a distinctive genetic profile, characterized by the overexpression of *KRT *genes and *TRIM29*, among others. Other subtypes were also characterized by the corresponding genes found in the Norway/Stanford data (Figure 5S and 6S in Additional file 1). Consistent with previous findings is the observation that the basal-like cluster is the most homogeneous in both Swedish cohorts, with a high correlation among samples and a clear separation from other subtypes.

### Consistency of clustering result

In the Swedish cohort, the overall concordance rate between the centroid prediction and the *k*-means clustering was 77.5% (Table 2), comparable to the 74.6% concordance rate observed for the Norway/Stanford data (data not shown). The highest rate of discordant assignments occurred between the luminal A and luminal B subtypes, and between the luminal B and ERBB2 subtypes. Thirty-four (9%) tumors were classified as luminal A by the centroid prediction but clustered as luminal B in the *k*-means clustering. A total of 21 (6%) tumors were classified as luminal B by the centroid prediction but clustered as ERBB2 in the *k*-means clustering. Twelve tumors had discordant assignments to the basal and ERBB2 subtypes. The normal-like subtype had the fewest discordant assignments.

To understand the reasons for discordant assignments, we investigated the expression of the distinguishing genes of each subtypes. The 21 luminal B tumors clustered to ERBB2 had relatively high expression of the *ERBB2 *and *GRB7 *genes and low expression of *ESR1 *gene, all characterizing the ERBB2 subtype. The 34 luminal A tumors clustered to luminal B displayed a high expression of *GGH *and *SQLE*, but low expression of *SCUBE2*, *NAT1 *and *LTF *[17]. In comparison with those consistently classified as basal-like (*n *= 46), the tumors clustered as ERBB2 (*n *= 12) showed a lower average expression in *KRT17*, *KRT5 *and especially *GABRP*, which has been reported to be associated with ER^-^/HER2^- ^breast cancers [18].

### Tumor subtypes and estrogen receptor status

Breast cancer subtype classification is closely related to ER status, with a high proportion of luminal A/B and normal-like tumors having ER-positive protein overexpression, whereas basal-like and ERBB2 tumors have a higher proportion of ER negativity [15,16,19]. These findings were confirmed in the Swedish data (Table 3). However, a large discrepancy was seen in the proportion of ER-positive tumors between the basal-like (45.8%) and ERBB2 (67.4%) groups. This discrepancy is surprising because we did not see any obvious difference in the expression of the ESR1 gene (Figure 1). For the luminal A/B and normal-like subtypes, the expression of the ESR1 gene seems consistent with the protein expression.

### Tumor subtypes and clinical characteristics

When the molecular subtypes were characterized according to tumor characteristics, treatment and HRT use, quite a complex pattern emerged (Table 4). The youngest women were found in the basal-like group, in which 27.5% were premenopausal. The average tumor size and the proportion of tumors smaller than 21 mm were not dramatically different among the groups, with the exception of the ERBB2 group, in which the tumor size tended to be larger. Basal-like tumors tended to have high Elston grade and to be genomically unstable, but surprisingly there was no significant difference in lymph-node metastasis status between the different subtypes. A majority (65%) of basal-like tumors had *p53*-sequence mutations and a relatively high 40% of the patients were current or former users of HRT.

The ERBB2 group consisted of elderly women with large tumors, of which 57% were Elston grade III, 39% had a *p53 *mutation and 71% were genomically unstable aneuploids, an even higher percentage than in the basal-like subtype. At the same time these patients were 67% ER-positive and 72% progesterone receptor-positive. The luminal B group revealed the same complex pattern as ERBB2 but on a less aggressive scale, particularly with a smaller proportion of unstable aneuploid tumors. Tumor size and receptor status indicated a low metastatic potential, yet 55% had an Elston grade of III.

Found mostly in postmenopausal women, the luminal A and normal-like tumors tended to be small and receptor positive, were unlikely to be Elston grade III, tended to have wild-type *p53 *status and tended to be genomically more stable. One striking difference between these two subtypes was in the ongoing use of HRT: 51% in the normal-like group versus 5% in luminal A.

### Tumor subtypes and prognosis

Genetically derived subtypes seem to be distinct biological entities, so we expect them to have prognostic implications. Overexpression of the *ERBB2 *gene has been associated with poor clinical outcome, whereas there is some indication of poor prognosis for tumors overexpressing citokeratin 17 and 5, characteristics of the basal-like subtype [16,20,21]. Previous findings [15] suggest that prognostic discrepancies between subtypes reflect different responses to therapy, especially endocrine therapies. To gain a better insight we performed separate analyses for patients with endocrine therapy and for those without any adjuvant treatment. However, interpretation is still rather limited as a result of the small number of patients.

Regardless of whether or not the patient received adjuvant therapy, the ERBB2 group had the worst recurrence-free survival, whereas the luminal A and normal-like groups had the best prognosis (Figure 2). Among endocrine-treated patients, the luminal B subtypes had a significantly lower survival than the luminal A and normal-like tumor groups, and the patients in the basal-like group did not differ significantly from those in the luminal A group. Among the untreated patients, however, the basal-like group had a survival pattern more similar to the luminal-B and ERBB2 patients. Similar results were obtained for breast cancer-specific survival (Figure 7S in Additional file 1).

## Discussion

We were able to confirm that the intrinsic signature described previously [1] exists in a broad population-based cohort of breast cancers. This is by far the largest validation and clinical characterization of the molecular subtypes to date. Previous characterization was given in [1] – *p53 *mutation status on 69 cases – and in an unpublished report [22], reviewed in [23]. The different prognosis of the subtypes was already reflected in the tumor characteristics, although the patterns were complex. By performing a separate analysis of endocrine-treated and adjuvant-untreated patients we were also able to evaluate the differences between subtypes in terms of both prognosis and response to therapy.

Normal-like tumors were small, found in postmenopausal women, seldom of Elston grade III, mostly stable diploid and more prevalent among HRT users. They had a favorable prognosis under endocrine therapy. Luminal-A tumors closely resembled normal-like tumors but were not associated with HRT use and had a superior prognosis regardless of adjuvant therapy. In contrast, the ERBB2 tumors showed a 50% relapse-free 5-year survival not influenced by therapy. The poor prognosis was reflected in large tumor size, high proportion of Elston grade III and unstable aneuploids, and few ER-positive tumors. Luminal B tumors had a clearly worse prognosis than luminal A ones, and were apparently less responsive to the hormonal therapy than the basal-like and normal-like tumors [2]. In comparison with the luminal A group, this group had a higher proportion of Elston grade III, p53 mutation and lymph-node positive tumours, confirming the hypothesis of a more proliferative profile [23]. Surprisingly, although the basal-like tumors had even fewer ER-positive tumors and more high-grade tumors, they had better relapse-free survival than the ERBB2 tumors, especially among the endocrine-treated patients. It is worth noting that about one-third of the patients with basal-like tumors used HRT at the time of diagnosis.

The intrinsic signature set of approximately 500 genes was originally selected on the basis of their stable expression between pairs of samples taken from the same tumors, before therapy and after 15 weeks of neoadjuvant therapy [1]. This implies that the set is enriched in genes whose expression patterns are characteristic for individual tumors as opposed to those that vary as a function of tissue sampling, and hence would be ideally suited for classification. Another recent study [24] showed that the subtypes were also observed among Asian-Chinese patients, although this study performed only the hierarchical clustering and identified only three subtypes.

The discordant subtyping between the centroid prediction and *k*-means clustering indicates some overlapping characteristics between subtypes, and potentially reveals a biological heterogeneity more complex than the subtypes alone. Discordances occurred most frequently between the luminal A and luminal B groups and between the luminal B and ERBB2 groups. Not surprisingly, despite differences in receptor status, luminal B and ERBB2 share similar clinical – such as tumor grade – and prognostic characteristics. This suggests that there must in fact be grade-associated and outcome-associated genes in the intrinsic gene set, and that these two subtypes have similar expression in these genes. A similar argument can also be made from the large confusion of luminal A and luminal B, namely that this must have been driven by ER-associated genes, because these two subtypes have similar receptor characteristics. Having the fewest discordances, the normal-like subtype seemed to have the most distinctive profile. One of the most striking characteristics was the high proportion of HRT users within this subtype.

Although the intrinsic signature is expected to reveal fundamental tissue properties, such as origin (basal-like versus luminal), our study supports the hypothesis that the subtypes are distinct biological entities with distinct clinical characteristics. Our study showed how they differed in progression-related characteristics, such as tumor grade, *p53 *mutation and genomic instability. This means that there must be a strong correlation between the intrinsic gene set and other progression-related signatures based on *p53 *transcription profile [4], proliferation or genomic instability, and possibly an inherent capability to metastasize [25]. The extent to which the different signatures contribute independent information toward clinical progression or prognosis requires further study.

Our findings further support the hypothesis of a reduced response to hormone-based treatments for ERBB2 and luminal B tumors [23]. Whereas luminal A tumors seem to have a good prognosis overall, luminal B tumors had a poor prognosis regardless of endocrine therapy, indicating some resistance to therapy. This may be explained at least partly by a worse clinical profile, with a higher tumor grading and a higher proportion of *p53 *mutation. In contrast, in the endocrine-treated group the basal-like subtype seemed to survive better than expected considering its poor clinical characteristics including its high proportion of Elston grade III (75%) and few ER-positive (46%) tumors. The ERBB2 tumors had the lowest relapse-free survival: approximately 50% of the patients had either died from breast cancer or experienced distant metastases. Overexpression of the human transmembrane tyrosine kinase growth factor receptor (HER-2), characteristic of the ERBB2 group, has been associated with more aggressive forms of tumor and with resistance to endocrine therapies [26-28].

## Conclusion

One strength of our study was that we were able to analyze hormone-adjuvant-treated and untreated patients separately. Thus, it seems that the molecular subtypes also carry therapy predictive information. However, when interpreting the results it should be emphasized that observational studies are not optimal for evaluating therapy. Reverse causality, in which a patient with a more aggressive tumor is more likely to receive therapy, is a major problem, producing an apparent result that therapy worsens survival. Ideally, to establish microarray-based predictive markers we would use a randomized comparison of adjuvant therapy versus placebo, but we are not currently aware of any such study.

Finally, from the clinical perspective, it is of highest importance to subgroup and characterize cancers so as to identify patients in need of aggressive therapy and those that could probably be spared adjuvant systemic therapy and thereby the adverse late health effects. We have been able to verify that the intrinsic signature classifies tumors into five groups with distinct tumor characteristics influencing relapse-free survival and probably also predictive of therapy response.

## Abbreviations

CMF = cyclophosphamide–methotrexate–5-fluorouracil; ER = estrogen receptor; HRT = hormone replacement therapy; SSI = stemline-scatter index.

## Competing interests

The authors declare that they have no competing interests.

## Authors' contributions

SC, YP and AP performed the biostatistical analyses and co-wrote the manuscript. PH and J Bergh co-wrote the manuscript. J Bjöhle contributed tumors, coordinated tissue and clinical data collection and the extraction of RNA for the Stockholm cohort. UK and GA collected data on genomic instability. SK, ETL and LM contributed tumors, coordinated the collection of tissue, and also clinical and microarray data for the Uppsala cohort. All authors read and approved the final manuscript.

## Supplementary Material

Additional file 1 A PDF file containing seven supplementary figures (Figures 1S – 7S). Click here for file

## Figures and Tables

**Table 1:** Norway/Stanford centroid size (*N*), minimum subtype correlation (*R*) and centroid-prediction assignments

Subtype	Norway/Stanford		Sweden	
	*N*	*R*	*N*	Percentage
Basal-like	17	0.37	59	14.3
ERBB2	7	0.23	43	10.4
Luminal A	9	0.27	122	29.6
Luminal B	7	0.20	54	13.1
Normal-like	7	0.35	91	22.1
Unclassified	-	-	43	10.4

Samples with a Pearson correlation of less than 0.1 were labeled as unclassified.

**Table 2:** Concordance between centroid-prediction classification (rows) and *k*-means clustering label assignment (columns) for the Swedish cohort

Subtype	Basal-like	ERBB2	Luminal A	Luminal B	Normal-like	Total
Basal-like	46	12	0	0	1	59
ERBB2	0	39	0	1	3	43
Luminal A	0	1	87	34	0	122
Luminal B	0	21	0	32	1	54
Normal-like	2	0	5	2	82	91
Total	48	73	92	69	87	369

**Table 3:** Estrogen receptor (ER) status by tumor subtype in the Swedish cohort

ER status	Basal-like	ERBB2	Luminal A	Luminal B	Normal-like
ER-negative	32	14	3	7	7
ER-positive	27	29	115	47	84
ER-positive (%)	45.8	67.4	97.5	87.0	92.3

**Table 4:** Descriptive statistics by tumor subtypes in the Swedish cohort

Characteristic	Basal-like	ERBB2	Luminal A	Luminal B	Normal-like	*p*
	*n *= 59	*n *= 43	*n *= 122	*n *= 54	*n *= 91	
Cohort (*n*)						0.51^e^
Stockholm	25	15	39	23	37	
Uppsala	34	28	83	31	54	
Mean age at breast cancer diagnosis	54	59	66	61	59	<0.001^e^
Menopausal status (%)						0.0807^e^
Premenopausal	27.5	13.3	10.9	24.4	18.6	
Postmenopausal	72.5	86.7	89.1	75.6	78.6	
Mean tumor size (mm)	22.9	27.9	21.8	22.6	20.7	0.0345^f^
Tumor size < 21 mm (%)	51	35	57	42	70	<0.001^e^
Elston grade						<0.001^e^
I	3.5	4.8	34.5	5.7	43.2	
II	21.1	38.1	54.3	39.6	52.3	
III	75.4	57.1	11.2	54.7	4.5	
*p53 *mutation (%)^a^	64.7	39.3	9.6	38.7	3.7	<0.001^e^
HRT (%)^b^						<0.001^e^
Never	60.0	78.6	81.6	82.6	48.6	
Former	8.0	7.1	13.2	8.7	0.0	
Ongoing	32.0	14.3	5.3	8.7	51.4	
Lymph-positive (%)	29	48	33	41	33	0.312^e^
Estrogen receptor positive (%)	45.8	67.4	97.5	87.0	92.3	<0.001^e^
Progesterone receptor positive (%)	54.2	72.1	93.2	77.8	92.3	<0.001^e^
Genomically unstable (%)^b^	76.0	78.6	56.4	78.3	37.1	0.0031^e^
Genomic instability by ploidy^b^						0.0123^e^
Diploid stable	12.0	7.1	25.6	4.3	40.0	
Diploid unstable	4.0	0.0	20.5	26.1	17.1	
Tetraploid stable	4.0	7.1	7.7	8.7	8.6	
Tetraploid unstable	16.0	7.1	12.8	13.0	0.0	
Aneuploid stable	8.0	7.1	10.3	8.7	14.3	
Aneuploid unstable	56.0	71.4	23.1	39.1	20.0	
Treatment type (%)^c^						0.0353^e^
None	39.7	35.7	43.3	35.8	46.2	
Endocrine	36.2	45.2	50.8	56.6	44.0	
Chemotherapy	15.5	16.7	5.0	5.7	7.7	
Radiotherapy	8.6	2.4	0.8	1.9	2.2	
Breast cancer event within 5 years (%)^d^	30	51	17	43	19	<0.001^e^

^a^*p53 *data were available only for the Uppsala cohort. ^b^HRT and genomic instability were available only for the Stockholm cohort. ^c^For the Stockholm cohort, endocrine comprises endocrine treatment alone and with any other combination. Chemotherapy refers to chemotherapy alone or associated with radiotherapy. Radiotherapy means radiotherapy alone. ^d^Breast cancer event refers to relapse or death for breast cancer. ^e^Pearson χ^2 ^test. ^f^Analysis of variance test.
