# Supplementary material for: Intrinsic molecular signature of breast cancer in a population-based cohort of 412 patients
Source: Breast Cancer Res. 2006 Jul 17;8(4):R34. doi: 10.1186/bcr1517 (PMC1779468; doi:10.1186/bcr1517)
Supplement: Additional file 1 — A PDF file containing seven supplementary figures (Figures 1S – 7S). [file bcr1517-S1.pdf]

Supplementary Report Intrinsic molecular signature of breast cancer in a population-based cohort of 412 patients

S Calza et al.

Figure 1S: Hierarchical clustering of Stanford/Norway data based on 516 genes. Dendrogram labels and colors correspond to subtype clusters.

Figure 2S: Gene profile for the five subtypes in the Stanford/Norway data.

Figure 3S: Stockholm hierarchical clustering based on 465 genes in common with the "Intrinsic set" in Sorlie et al**?**. Labels and colors correspond to centroid-predicted assignment of tumor subtype.

Figure 4S Uppsala hierarchical clustering based on 465 genes in common with the "Intrinsic set" in Sorlie et al**?**. Colors correspond to centroid-predicted assignment of tumor subtype.

Figure 5S: Gene profile for some subtypes in the Stockholm cohort. Labels indicate the centroid label whose the sample is more correlated to and the Estrogen Receptor status.

Figure 6S: Gene profile for some subtypes in the Uppsala cohort. Labels indicate the centroid label whose the sample is more correlated to and the Estrogen Receptor status.

Figure 7S: Kaplan-Meier breast-cancer specific survival curves of the tumor subtypes in (a) Sweden with endocrine therapy, (b) Uppsala without any adjuvant therapy. The P-value, computed in a Cox regression, is for simultaneous comparison of all the curves.

Figure 1S:

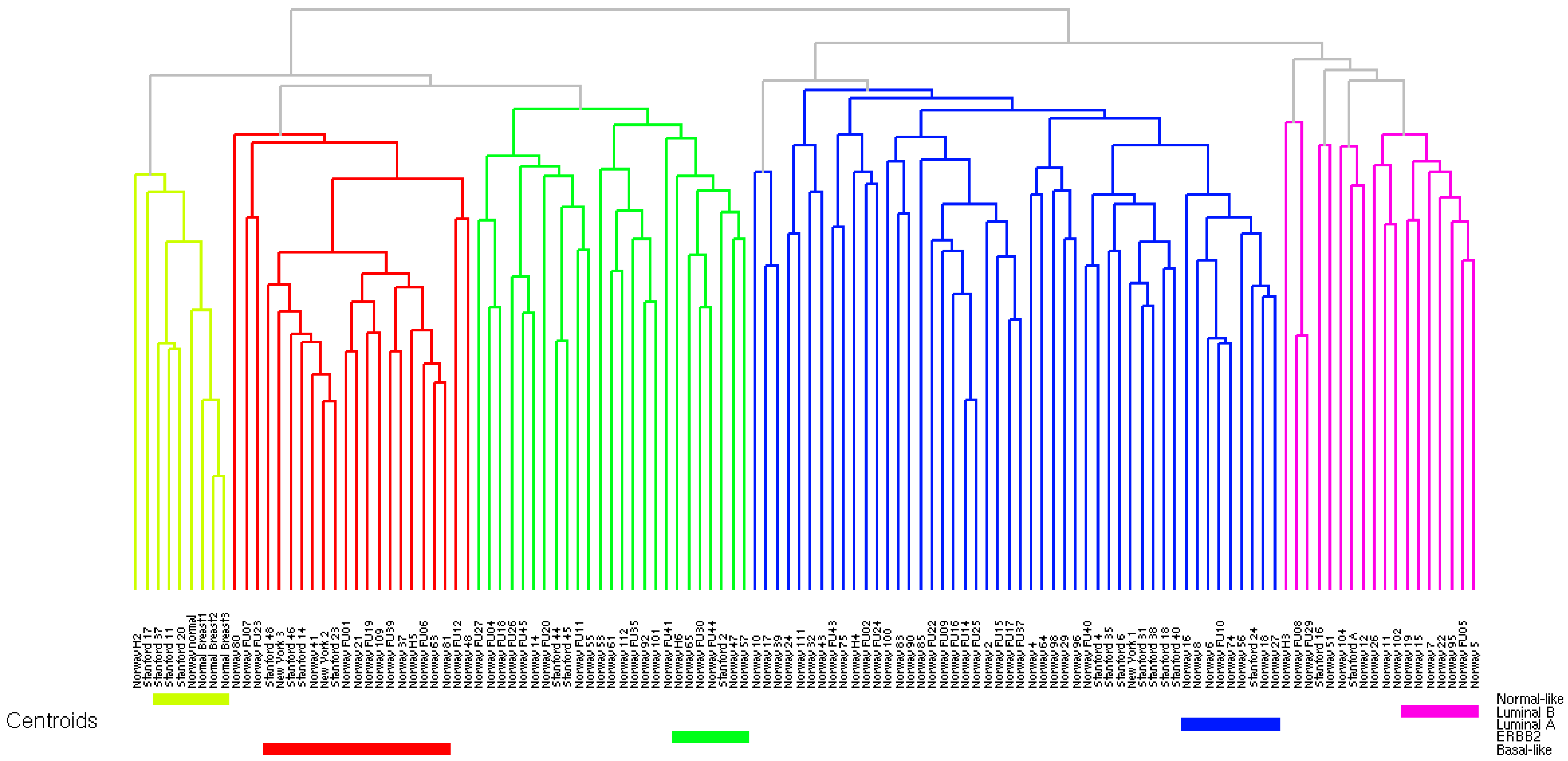
Norway H2
Stanford 17
Stanford 37
Stanford 11
Stanford 20
Norway normal
Normal Breast1
Normal Breast2
Normal Breast3
Norway 80
Norway FU07
Norway FU23
Stanford 48
New York 3
Stanford 46
Stanford 14
Norway 41
New York 2
Stanford 23
Norway FU01
Norway 21
Norway FU19
Norway 109
Norway FU39
Norway 37
Norway H5
Norway FU06
Norway 63
Norway 81
Norway FU12
Norway 48
Norway FU27
Norway FU04
Norway FU18
Norway FU26
Norway FU45
Norway 14
Norway FU20
Stanford 44
Stanford 45
Norway FU11
Norway 55
Norway 53
Norway 61
Norway 112
Norway FU35
Norway 92
Norway 101
Norway FU41
Norway H6
Norway 65
Norway FU30
Norway FU44
Stanford 2
Norway 47
Norway 57
Norway 10
Norway 17
Norway 39
Norway 24
Norway 111
Norway 32
Norway 43
Norway FU43
Norway 75
Norway H4
Norway FU02
Norway FU24
Norway 100
Norway 83
Norway 90
Norway 85
Norway FU22
Norway FU09
Norway FU16
Norway FU14
Norway FU25
Norway 2
Norway FU15
Norway FU17
Norway FU37
Norway 4
Norway 64
Norway 98
Norway 29
Norway 96
Norway FU40
Stanford 4
Stanford 35
Stanford 6
New York 1
Stanford 31
Stanford 38
Stanford 18
Stanford 40
Norway 16
Norway 8
Norway 6
Norway FU10
Norway 74
Norway 56
Stanford 24
Norway 18
Norway 27
Norway H3
Norway FU08
Norway FU29
Stanford 16
Norway 51
Norway 104
Stanford A
Norway 12
Norway 26
Norway 11
Norway 102
Norway 19
Norway 15
Norway 7
Norway 22
Norway 95
Norway FU05
Norway 5
Centroids
Normal-like
Luminal B
Luminal A
ERBB2
Basal-like

Figure 2S:

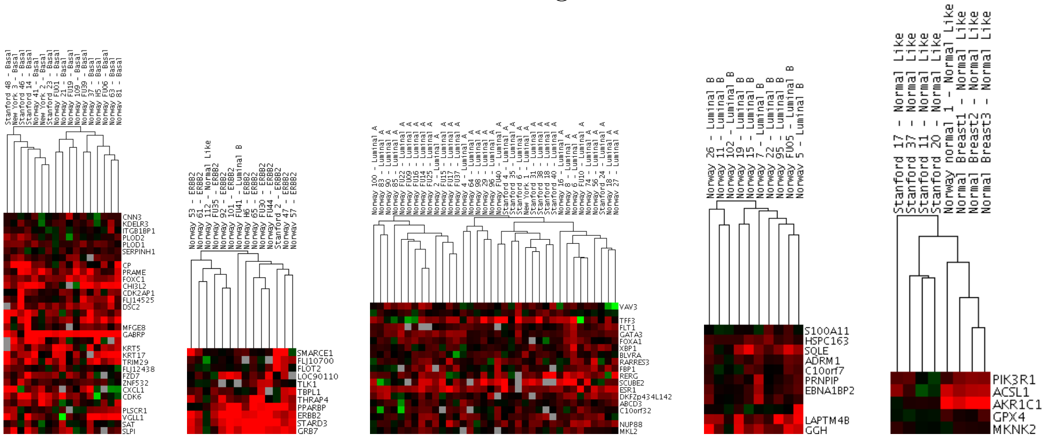

Basal-like ERBB2 Luminal A Luminal B Normal-like

Figure 3S:

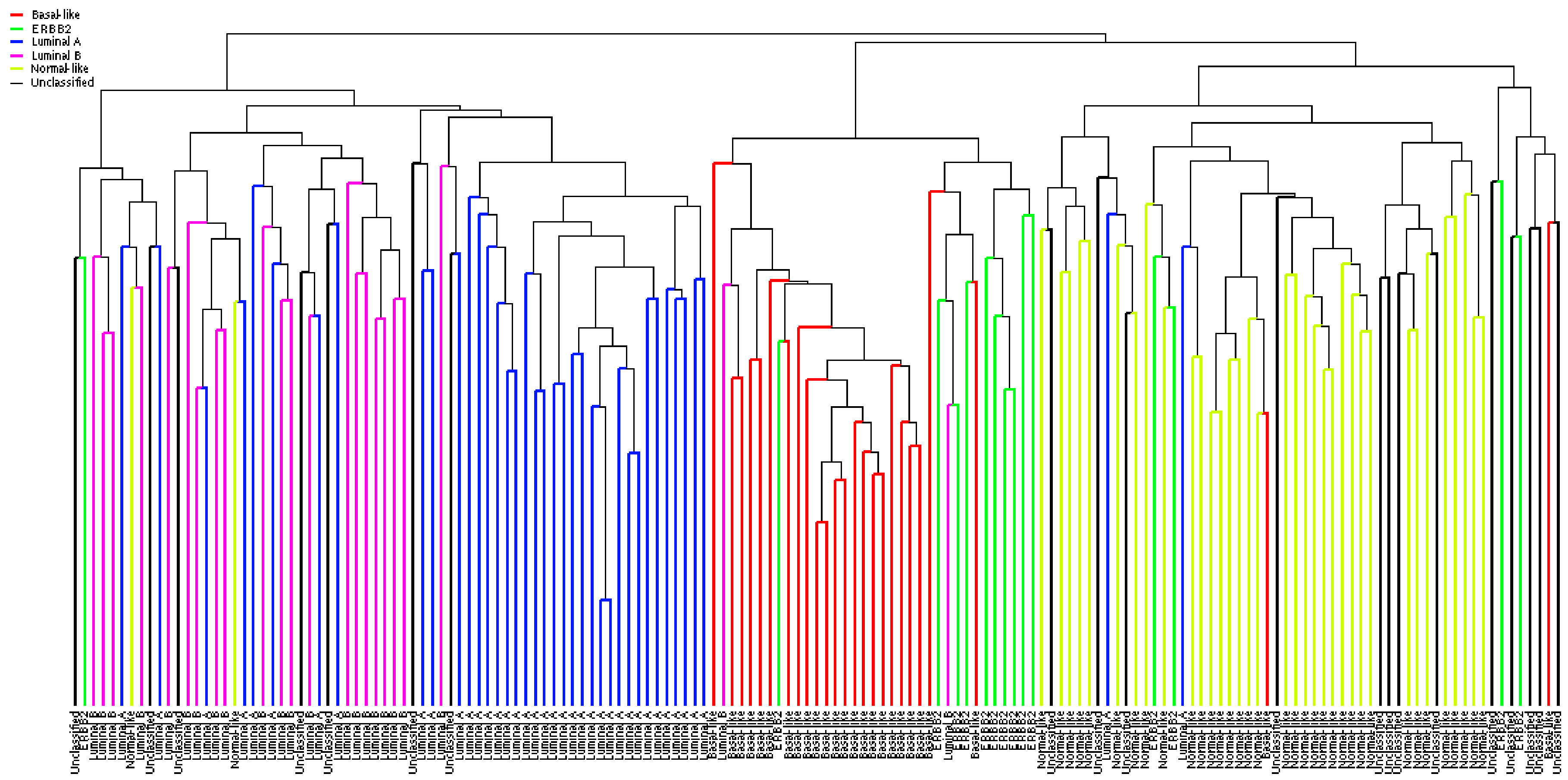

Basal-like
ERBB2
Luminal A
Luminal B
Normal-like
Unclassified

Figure 4S:

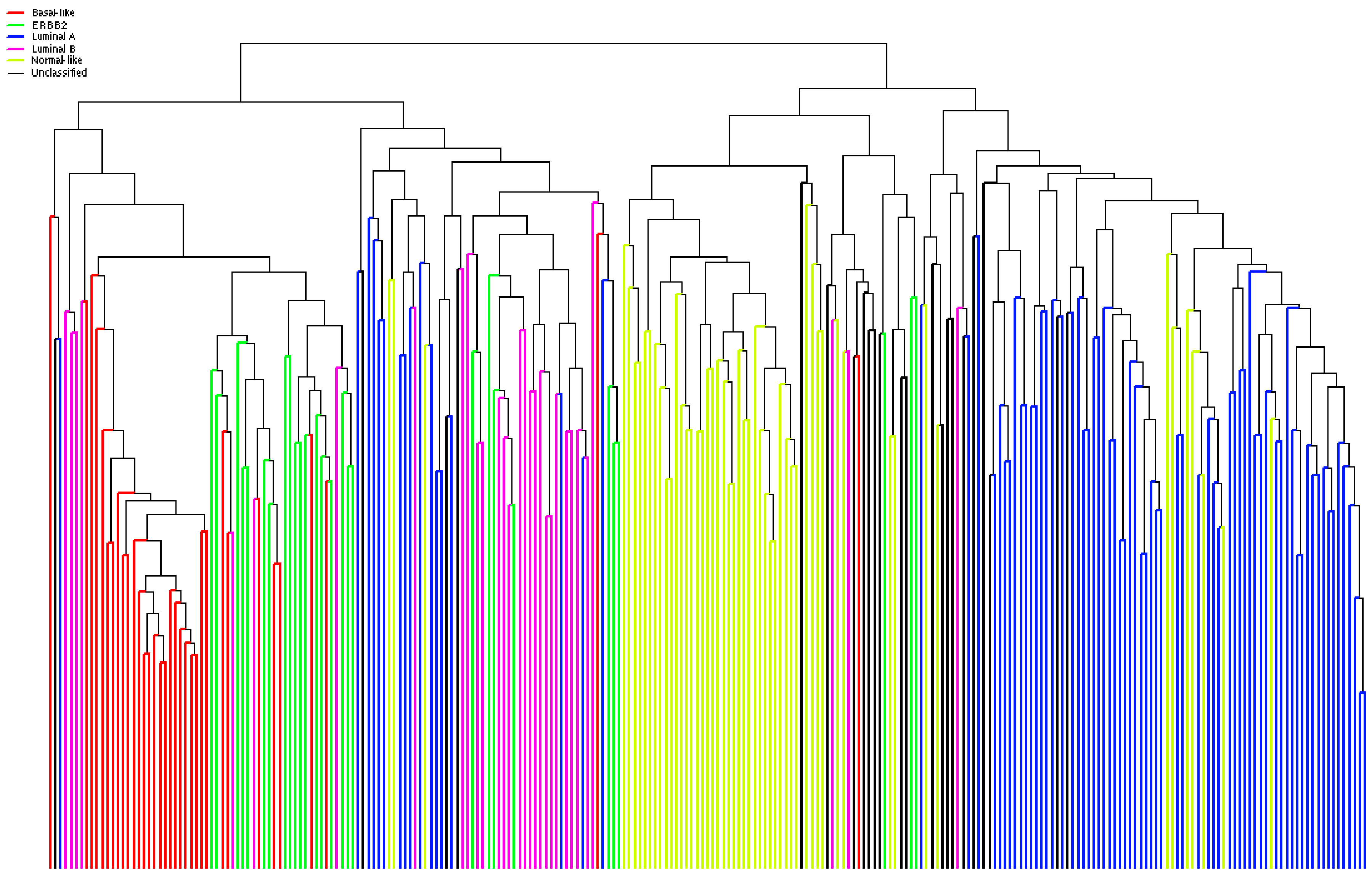
Basal-like
ERBB2
Luminal A
Luminal B
Normal-like
Unclassified

Figure 5S:

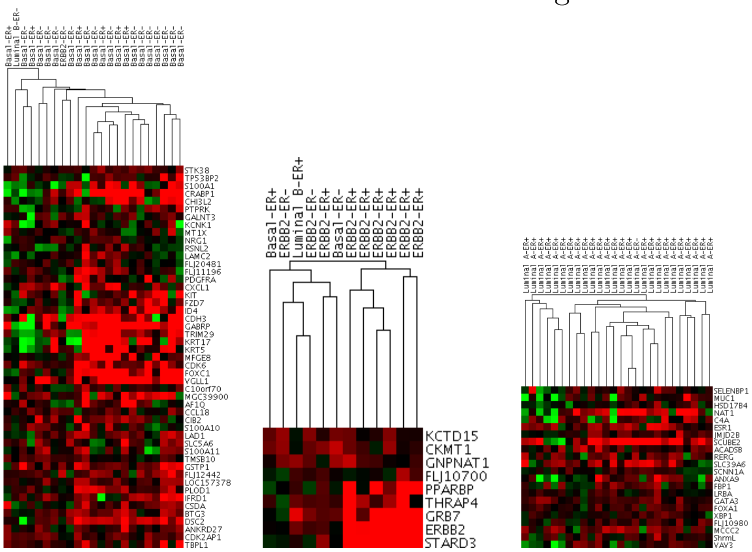

(a) Basal-like (b) ERBB2 (c) Luminal A

Figure 6S:

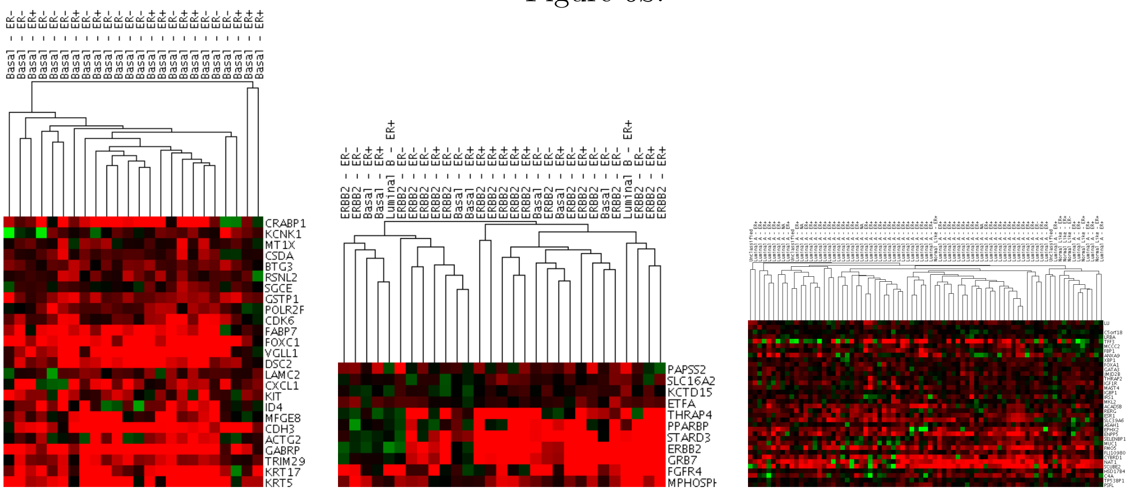

(a) Basal-like (b) ERBB2 (c) Luminal A

Figure 7S:

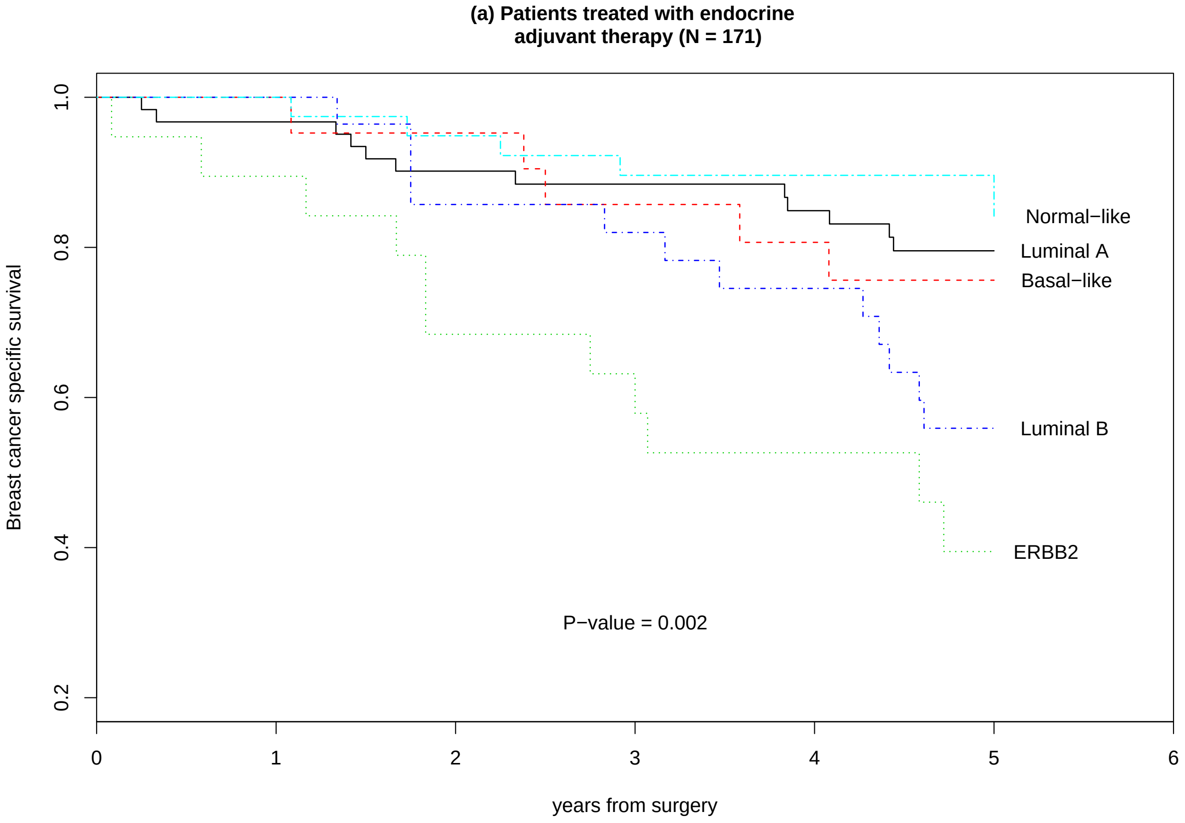

(a) Patients treated with endocrine adjuvant therapy (N = 171)
Breast cancer specific survival
0.2
0.4
0.6
0.8
1.0
0
1
2
3
4
5
6
years from surgery
Normal−like
Luminal A
Basal−like
Luminal B
ERBB2
P−value = 0.002

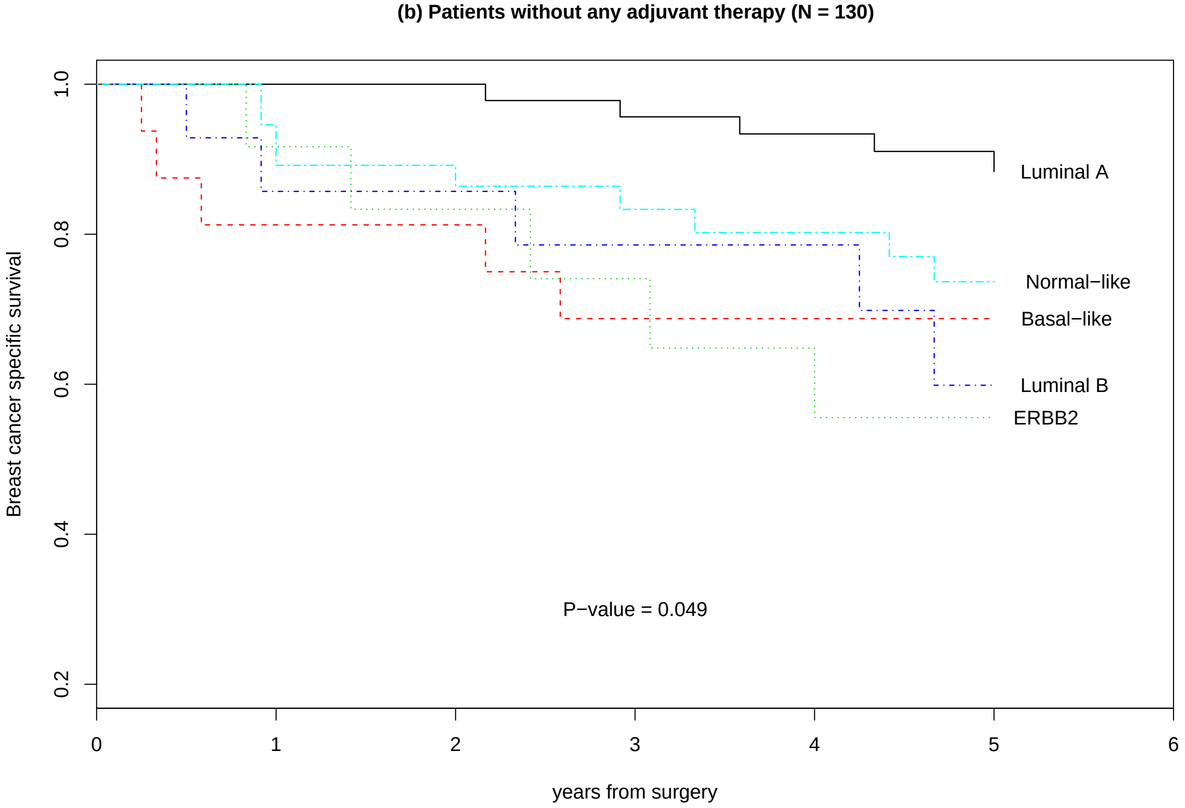

(b) Patients without any adjuvant therapy (N = 130)
Breast cancer specific survival
0.2
0.4
0.6
0.8
1.0
0
1
2
3
4
5
6
years from surgery
Luminal A
Normal−like
Basal−like
Luminal B
ERBB2
P−value = 0.049
